# SARS-CoV-2 Specific IgG Antibodies Persist Over a 12-Month Period in Oral Mucosal Fluid Collected From Previously Infected Individuals

**DOI:** 10.3389/fimmu.2021.777858

**Published:** 2021-12-09

**Authors:** Prithivi Chellamuthu, Aaron N. Angel, Melanie A. MacMullan, Nicholas Denny, Aubree Mades, Marilisa Santacruz, Ronell Lopez, Cedie Bagos, Joseph G. Casian, Kylie Trettner, Lauren Lopez, Nina Nirema, Matthew Brobeck, Noah Kojima, Jeffrey D. Klausner, Fred Turner, Vladimir Slepnev, Albina Ibrayeva

**Affiliations:** ^1^ Department of Serology Research and Development, Curative, Monrovia, CA, United States; ^2^ Mork Family Department of Chemical Engineering and Materials Science, Viterbi School of Engineering, University of Southern California, Los Angeles, CA, United States; ^3^ Department of Medicine, University of California, Los Angeles, Los Angeles, CA, United States; ^4^ Department of Population and Public Health Sciences, Keck School of Medicine, University of Southern California, Los Angeles, CA, United States; ^5^ Eli and Edythe Broad Center for Regenerative Medicine at the University of Southern California, William Myron Keck School of Medicine, Los Angeles, CA, United States; ^6^ Davis School of Gerontology, University of Southern California, Los Angeles, CA, United States

**Keywords:** SARS-CoV-2 antibodies, oral mucosal fluid immunity, SARS-CoV-2 immunology, antibody monitoring, ELISAs

## Abstract

**Background:**

Developing an understanding of the antibody response, seroprevalence, and seroconversion from natural infection and vaccination against SARS-CoV-2 will give way to a critical epidemiological tool to predict reinfection rates, identify vulnerable communities, and manage future viral outbreaks. To monitor the antibody response on a larger scale, we need an inexpensive, less invasive, and high throughput method.

**Methods:**

Here we investigate the use of oral mucosal fluids from individuals recovered from SARS-CoV-2 infection to monitor antibody response and persistence over a 12-month period. For this cohort study, enzyme-linked immunosorbent assays (ELISAs) were used to quantify anti-Spike(S) protein IgG antibodies in participants who had prior SARS-CoV-2 infection and regularly (every 2-4 weeks) provided both serum and oral fluid mucosal fluid samples for longitudinal antibody titer analysis.

**Results:**

In our study cohort (n=42) with 17 males and 25 females with an average age of 45.6 +/- 19.3 years, we observed no significant change in oral mucosal fluid IgG levels across the time course of antibody monitoring. In oral mucosal fluids, all the participants who initially had detectable antibodies continued to have detectable antibodies throughout the study.

**Conclusions:**

Based on the results presented here, we have shown that oral mucosal fluid-based assays are an effective, less invasive tool for monitoring seroprevalence and seroconversion, which offers an alternative to serum-based assays for understanding the protective ability conferred by the adaptive immune response from viral infection and vaccination against future reinfections.

## Introduction

As of August 2021, the novel coronavirus, SARS-CoV-2, has had a detrimental global impact with over 200 million reported cases, 4.4 million lives lost, and economic calamities worldwide (1). Technological breakthroughs in vaccine development and mass vaccinations in countries like the United States and Israel are proving effective for case management and mitigation of its impacts (2). Despite the early successful efforts in controlling SARS-CoV-2 infection, the viral variants have remained within the population with a likelihood of developing into an endemic disease. Additional research is needed to understand seroprevalence, seroconversion, the persistence of antibody against the virus, the antibody titers in naturally infected *versus* vaccinated population, and the clinical implications related to immunity offered.

Long-term humoral immunity is mediated by various classes of antibodies. The trajectories of the development and decay of commonly described antibodies IgA, IgM and IgG, experience independent peaks and only overlap during early periods (less than one month post exposure). The concentrations of IgM and IgA antibodies diminish too quickly to conduct long-term studies, typically within a month of infection (3, 4). However, IgG concentrations, specifically for SARS-CoV-2, remain high and stable even after several months (5) and seem to correlate with concentrations of neutralizing antibody titers (6). For these reasons, IgG is an extremely valuable biomarker for tracking long-term immune responses.

Humoral immune response monitoring *via* antibody titer levels using automated, high-throughput ELISAs offers an accurate, and scalable method to survey the prevalence of antibodies in a population. Current serum-based ELISAs have several limitations including invasiveness of specimen collection, higher cost, required assistance of a health care worker, and advanced sample processing. To effectively monitor seroconversion and seroprevalence within a population, an effective, and non-invasive method for antibody detection is required. Oral-fluid based assays could act as proxy to serum-based assays, as they have been successfully used to detect or monitor antibody levels for other clinical conditions such as HIV infection, Hepatitis C, Measles, and Rubella (7–9). To that end, OraSure Technologies^®^ has developed an oral specimen collection device (OSCD) and a total antibody ELISA for use with oral mucosal fluid collected from this device. An earlier study from our group showed that it is possible to quantify the antibody titers from oral mucosal fluids collected by the OSCD (10). It should be noted that, at present, the relationship between concentrations of antibody and possible immune protection is not well understood.

From the perspective of monitoring long-term humoral immunity, the question of how long we can expect antibodies against the novel SARS-CoV-2 virus to persist both in serum and oral mucosal fluid remains. Thus, we have designed and conducted a longitudinal clinical study to further understand the relationship between SARS-CoV-2 infection and the persistence and change in IgG antibody titers over time to advance our understanding and its potential implication for long-term immunity. Here, we collected, analyzed, and quantified SARS-CoV-2 IgG in oral mucosal fluids and serum of individuals at various timepoints over a period of one year and focus on the persistence of IgG antibody levels in oral mucosal fluids.

## Materials and Methods

### Study Design

Participation was offered to subjects who were 18 years of age and tested negative or positive for COVID-19 *via* PCR test on oral swab specimens at a Curative site in Los Angeles County. Enrollment aimed for 240 participants with at least 1/3 negative, to be used as controls, and 2/3 positive by PCR, including 30% asymptomatic positive participants. Once enrolled in the study, participants may unenroll at any time for any reason. Participants were compensated $50 per visit and began the study approximately 14 days after symptom-offset to ensure the safety of the phlebotomists. Collections were scheduled once every 7 days for the first month, twice a month for the following month, and once a month thereafter. The actual participant adherence followed as 10-14 days between each appointment for the first six collections.

Previous studies have indicated that the highest SARS-CoV-2 viral load occurs two days before the onset of symptoms (11), thus SARS-CoV-2 IgG, hereafter IgG, dynamics were analyzed in relation to days post-symptom onset (PSO). Oral mucosal fluid sample availability for this study was limited due to preliminary assay optimization and lower sample volume compared to serum. Due to this limitation, we were not able to obtain IgG concentrations for every time point for all participants.

### Oral Mucosal Fluid IgG Longitudinal Study Cohort

For the current study, we included individuals who met the following criteria: 1) were originally confirmed SARS-CoV-2 positive by PCR assay; 2) had at least three oral mucosal fluid samples available for testing throughout the study period of approximately 12-months; 3) reported a symptom onset date; and 4) reported specific symptoms that they experienced. Symptomology of participants following infection was recorded at the time of first sample collection. Participants reported various flu-like symptoms, including fever, cough, chills, shortness of breath, chest pain/tightness, diarrhea, stuffy nose, sore throat, muscle pain, headache, weakness, as well as other SARS-CoV-2 specific symptoms including loss of taste and smell.

### Oral Mucosal Fluid IgG Post-Vaccination

A subset of the participants received COVID-19 vaccination (Pfizer or Moderna) toward the end of the study which provided us a means to understand vaccine induced IgG response in previously infected individuals.

### Human Oral Mucosal Fluid and Serum Sample Collection

Oral mucosal fluid samples were self-collected using the OraSure Technologies oral specimen collection device (OSCD) (OraSure^®^ Technologies, Bethlehem, PA). The collection was supervised by the same phlebotomist that performed the serum collection, the tip was broken off the collection device and the device were then placed into a secondary tube. For sample processing at the laboratory, the secondary tubes were centrifuged at 800 RCF for 15 minutes. When finished, the collection device was disposed of in a biohazard bag and the solution eluted from the collection pad was aliquoted into labeled microcentrifuge tubes and stored at -80°C until assay.

For serological sample collection, participants undergo a standard venipuncture procedure. Licensed phlebotomists sampled approximately 10 mL of blood using a 21G straight needle with a safety cap (BD; Franklin Lakes, NJ) into two Serum Separation Transport tubes (BD; Franklin Lakes, NJ). Once collected, the sample remained at ambient temperature for 60 minutes to coagulate and was then centrifuged at 1000 RCF for 15 minutes. Samples were then placed on ice until delivered to the laboratory site where the serum was aliquoted and stored at -80°C until assay.

### PCR Self-Sampling

The RT-PCR SARS-CoV-2 test (FDA-EUA 137809) is healthcare worker-observed, self-administered and proceeds according to previously reported guidelines (12). Briefly, participants cough hard three times while shielding their cough *via* mask and/or coughing into the crook of their elbow and then proceed to swab, in the following order, the inside of their cheeks, along the top and bottom gums, under the tongue, and finally on the tongue, to gather enough saliva.

### Oral Mucosal Fluid-Based Antibody Tests

The OraSure Technologies SARS-CoV-2 Total Antibody saliva-based assay (OraSure^®^ Technologies, Bethlehem, PA) binds antibodies that target both S1 and S2 subunits of the SARS-CoV-2 spike protein, the mediator in cell entry and infection. The OraSure assay requires the use of oral mucosal fluid collected from the OSCD which contains a preservative. The automated assay sequence, using the Dynex DSX Automated ELISA system (Dynex Technologies; Chantilly, VA), is as follows: a) Prior to sample loading, 25uL of a diluent solution is first added to each well followed by 100 µL of sample; b) The sample is then incubated for one hour at ambient temperature. The DSX houses a plate washer that washes each strip individually using a time delay function adjusted for the time it takes to add the samples to the wells. Each well was washed 6 times with 300 µL of wash buffer; c) 100 µL of the peroxidase-conjugated rabbit anti-human IgG (H+L) enzyme conjugate was added to each well and incubated for one hour at ambient temperature; d) Following another six well washes, 100 µL of enzyme substrate was added to each well and allowed to incubate for 30 minutes. The final addition of 100 µL of stopping solution quenches the reaction; e) Absorbance values of the samples on the plate are then read on the DSX plate reader both at 450nm and 620nm. The final absorbance signal represents IgG levels in the sample, adjusted for background (620nm). All oral mucosal fluid samples were run in duplicate and the average of the absorbances were taken for analysis.

### Quantification of IgG Titers in Oral Mucosal Fluids

The antibody assay was performed according to the manufacturer’s instructions. The OraSure kit includes a calibrator, negative control, and a positive control that was added to every plate. All the samples and runs were qualified as per manufacturer’s recommendation. The ratio of the absorbance of each sample to the calibrator average was taken and compared to the manufacturer’s suggested cutoff values. The manufacturer recommended cutoff values for determination of samples were <0.8 for the negative samples and >1.0 for the positive samples. Samples in the range of 0.8 to 1.0 were considered equivocal but were still utilized in the quantification portion of the study.

Relative quantification of antibody titer was performed using a S1-specific monoclonal IgG antibody as a reference antibody, which had no known cross-reactivity to the S2 domain of the spike protein. Additionally, we did not observe cross reactivity of IgA and IgM antibodies (monoclonal) in our IgG quantification assay. The standard curve was used to calculate the IgG antibody concentration in specimens from absorbance values at 450/630 nm from the ELISA assay. Specimens with antibody titer levels exceeding the range of the standard curve were diluted in a sample dilution buffer and re-ran. The Limit of Detection (LOD) was 1 ng/mL and the Limit of Quantification (LOQ) was 1.5 ng/mL.

### Quantification of IgG Titers in Serum

EuroImmun (Catalog #: EI2606-9601 G, Lubeck, Germany) SARS-CoV-2 IgG ELISA targeting the S1 subunit of the spike protein was performed according to a published protocol (13) on the Thunderbolt automated instrument (Gold Standard Diagnostics (GSD); Davis, CA). Briefly, sera were diluted 1:101 in each well with buffer and then incubated at 37°C for 1 hour. Sample wells were washed three times, then conjugate was added and incubated at 37°C for 30 minutes. After another wash step as previously described, the substrate was added and incubated at ambient temperature for 30 minutes. Stopping solution was then added and the absorbance of sample wells was measured immediately at both 450 nm and 600 nm. The reading at 600 nm was automatically subtracted as noise during output report generation. All samples were run in duplicate, and the data was exported and analyzed according to established protocol (13) to obtain a ratio (Equation 1) for the adjusted optical density of the sample well. This ELISA kit itself was developed specifically for detection of SARS-CoV-2 IgG antibodies specific to the S1 subunit of the spike protein in serum. The quantification method was developed using a monoclonal IgG1 antibody specific for the S1 subunit of the SARS-CoV-2 spike protein from (Catalog#:NR-52392, BEI Resource, Manassas, VA) The stock concentration is 1.04 mg/mL and a standard curve with five dilutions was created at 3.1, 6.25, 12.5, 25, and 30 ng/mL.


$$Ratio=\frac{OD\;of\;the\;control\;or\;clinical\;sample}{OD\;of\;the\;calibrator\;average}$$

Equation 1. Antibody detection is qualified by the ratio, determined by dividing the OD of the control or sample by the averaged OD of the test kit calibrator.

### Data Analysis

All statistical analysis, and plots were generated using GraphPad Prism Software (GraphPad Prism, San Diego, USA). Correlation, ANOVA, t-test analyses were done on datasets using built-in functions of the Prism software. ANOVA and paired t-tests were chosen to compare and identify significance in changes in antibody concentration over time in individual participants.

### Ethical Approval

The study was initially approved by The UCLA Institutional Review Board (UCLA IRB) (IRB#20-000703). The UCLA IRB waived the requirement for signed informed consent for the research under 45 CFR 46.117(c) (2). The study team complied with all UCLA and Advarra IRB policies and procedures, as well as with all applicable Federal, State, and local laws regarding the protection of human subjects in research as stated in the approved IRB (Pro00045766).

## Results

**Figure 1:**
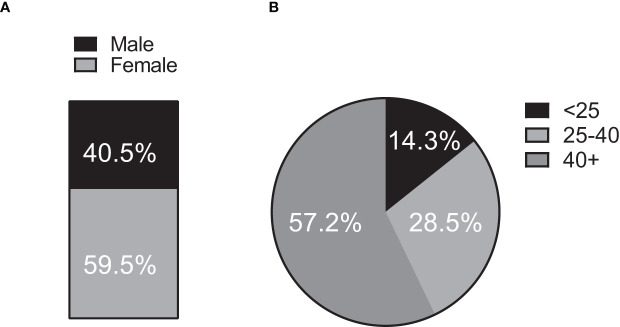
Population demographics. 42 (n = 42) individuals contributed oral mucosal fluid samples at timepoints spanning over 12-months for the clinical study. **(A)** Sex representation. 17 of 42 participants (40.5%) identified as male and 25 (59.5%) as female. **(B)** Age representation. Seven (7) individuals (16.6%) were younger than 25 years of age at the time of sample collection, 11 (26.2%) were between the ages of 25 and 40 and 24 individuals (57.2%) were older than 40 years of age.

From April 2020 to May 2021 a total of 339 participants were enrolled. During the study, 136 participants were unenrolled due to incomplete sample return. 42 PCR positive participants who experienced symptoms following infection were selected for this analysis. Of the 42 participants, 17 individuals (40.5%) identified as male and 25 (59.5%) identified as female (**Figure 1A** and **Table 1**). The average age of participants was 45.5 +/- 19.3 years and age bins for categorization and analysis were selected based on previous studies (14). During the time of collection, 7 individuals (16.6%) were younger than 25 years of age, 11 (26.2%) were between the ages of 25 and 40, and 24 individuals (57.2%) were older than 40 years of age (**Figure 1B** and **Table 1**).

**Table 1:** Summary of oral mucosal fluid and serum samples collected from each participant, including their age and sex.

Participant	Age	Sex	Sample Type	Timepoints(Total)
S1	58	F	Serum	10
S2	19	F	Serum	10
			Oral Fluid	7
S3	62	F	Serum	10
S4	25	F	Serum	9
			Oral Fluid	6
S5	59	M	Serum	9
S6	54	M	Serum	10
S7	50	F	Serum	9
			Oral Fluid	6
S8	25	F	Serum	9
S9	22	M	Serum	8
S10	27	F	Serum	10
			Oral Fluid	6
S11	58	F	Serum	9
			Oral Fluid	5
S12	40	M	Serum	10
			Oral Fluid	9
S13	43	F	Serum	8
			Oral Fluid	4
S14	27	F	Serum	5
			Oral Fluid	5
S15	22	F	Serum	9
S16	22	F	Serum	9
			Oral Fluid	8
S17	37	F	Serum	8
S18	43	F	Serum	9
			Oral Fluid	10
S19	31	M	Serum	8
S20	56	F	Serum	10
			Oral Fluid	7
S21	48	M	Oral Fluid	7
S22	25	F	Serum	10
			Oral Fluid	5
**Participant**	**Age**	**Sex**	**Sample Type**	**Timepoints (Total)**
S23	24	F	Serum	9
S24	36	M	Serum	10
S25	41	F	Serum	9
			Oral Fluid	8
S26	23	M	Serum	10
			Oral Fluid	6
S27	37	M	Serum	9
			Oral Fluid	3
S28	42	M	Oral Fluid	7
S29	49	F	Serum	11
			Oral Fluid	11
S30	30	M	Oral Fluid	9
S31	22	M	Oral Fluid	10
S32	36	F	Serum	10
			Oral Fluid	8
S33	42	M	Serum	10
			Oral Fluid	9
S34	22	M	Serum	10
			Oral Fluid	8
S35	57	F	Serum	9
			Oral Fluid	9
S36	50	F	Serum	9
			Oral Fluid	8
S37	39	M	Serum	10
S38	30	M	Serum	10
			Oral Fluid	5
S39	22	M	Serum	10
			Oral Fluid	8
S40	22	F	Oral Fluid	9
S41	25	F	Oral Fluid	8
S42	26	F	Oral Fluid	5
S43	62	F	Oral Fluid	8
S44	66	M	Oral Fluid	7
S45	66	F	Oral Fluid	9
S46	78	M	Oral Fluid	7
S47	69	F	Oral Fluid	7
**Participant**	**Age**	**Sex**	**Sample Type**	**Timepoints (Total)**
S48	75	F	Oral Fluid	8
S49	67	F	Oral Fluid	7
S50	69	F	Oral Fluid	7
S51	69	M	Oral Fluid	6
S52	74	M	Oral Fluid	8
S53	70	F	Oral Fluid	6
S54	71	M	Oral Fluid	6
S55	69	M	Oral Fluid	7

**Figure 2:**
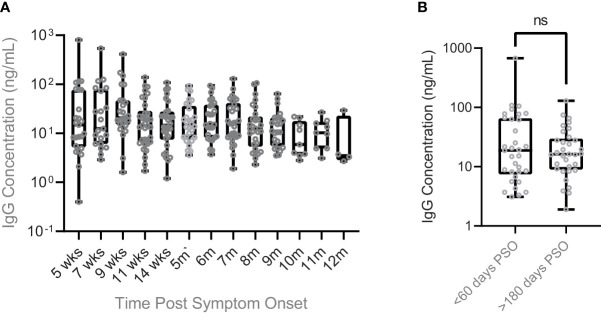
Persistence of SARS CoV-2 IgG antibody levels in oral mucosal fluids of COVID infected individuals. **(A)** SARS-CoV-2 IgG antibody concentrations were maintained for up to a year post symptom onset. Individuals who received vaccination during the time of this clinical study had their samples following vaccination omitted from this plot. The average antibody concentration was 62.64 +/- 159.3 ng/mL after 5 weeks PSO and 9.750 +/- 13.04 ng/mL after 12 months PSO. **(B)** A comparison of SARS-CoV-2 antibody concentration in the early days post symptom onset (<60 days) to the later days (>180 days) finds nonsignificant change in concentration over time. The average concentration of early SARS-CoV-2 IgG antibodies collected fewer than 60 days post symptom onset was 51.32 +/- 116.3 ng/mL, while after more than 180 days post symptom onset the average concentration dropped to 25.17 +/- 25.99 ng/mL. A paired two-sample t-test comparing these means found the decrease to be non-significant (p = 0.1915). ns, not significant.

IgG antibody response to SARS-CoV-2 infection from post symptom onset (PSO) was quantified in oral fluid specimens over a period of 12 months. The average IgG titers remained relatively stable over a period of nearly one year (**Figure 2A**). A paired t-test comparison of IgG concentrations in oral mucosal fluid samples collected in the first 2 months following symptom onset (51.32 +/- 116.3 ng/mL) and after 6 months post symptom onset (25.17 +/- 25.99 ng/mL) did not find a statistically significant decline (p = 0.1915) (**Figure 2B**). Antibodies remained detectable in all participants for up to 12 months following symptom onset, and the average concentration of the most recent timepoint (365 +/- 30 days) for all individuals was 9.75 +/- 13.04 ng/mL.

**Figure 3:**
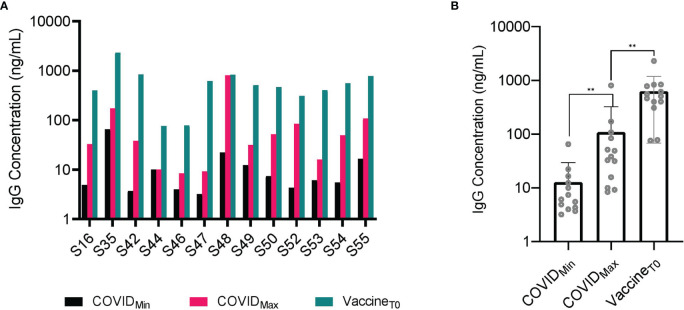
Vaccinated individuals experienced a significantly higher concentration of SARS-CoV-2 IgG antibodies in oral mucosal fluid than that prompted by viral infection. **(A)** SARS-CoV-2 IgG antibody concentration in fourteen (n = 14) individuals who received vaccination following infection. All 14 individuals experienced a significant (p-value < 0.001) increase in SARS-CoV-2 IgG antibody concentration following vaccination by paired two sample t-test. **(B)** A comparison between minimum (COVID_Min) and maximum antibody (COVID_Max) concentration post infection with antibody concentration post vaccination (Vaccine T0) reveals a statistically significant increase by paired two-sample t-test (p-value <0.001). Even compared to the highest concentration of post infection SARS-CoV-2 antibodies in these individuals at an average concentration (107 +/-206 ng/mL), the average concentration of post vaccination SARS-CoV-2 antibodies (626 +/- 537 ng/mL) was significantly higher. **p-value < 0.001.

While our primary focus was to characterize the persistence of IgG in oral mucosal fluid samples over a period of one year, a small number (n=13) of the study participants received vaccination during the study (**Table 2**). These participant specimens collected after vaccination were excluded from the longitudinal and correlation analyses. We looked at the individuals’ mean IgG antibody concentration at the first time point (Vaccine T0, 626.9 +/- 559.1 ng/mL) post vaccination and found that it was higher than the maximum (COVID_Max,_ 108.8 +/- 214.2 ng/mL) observed endogenous IgG concentration (**Figures 3A, B**).

**Table 2:** Summary of total oral mucosal fluid collections from the vaccinated participants of the study.

Participant	Age	Sex	Vaccine Manufacturer	Time Elapsed from 1st dose	Time points (Total)
S16	22	F	N/A	8 days	9
S35	56	F	Pfizer	9 days	10
S42	26	F	Pfizer	0 days	7
S44	66	M	Pfizer	6 days	8
S46	78	M	Moderna	32 days	8
S47	69	F	Moderna	18 days	8
S48	75	F	Moderna	34 days	10
S49	67	F	Moderna	37 days	9
S50	69	F	Moderna	28 days	8
S52	74	M	Moderna	44 days	9
S53	70	F	Pfizer	30 days	7
S54	71	M	Pfizer	30 days	7
S55	69	M	Moderna	39 days	9

Vaccine manufacturer, time elapsed from their first dose to their maximum IgG concentration, as well as sex and age are described.

NA, not applicable.

**Figure 4:**
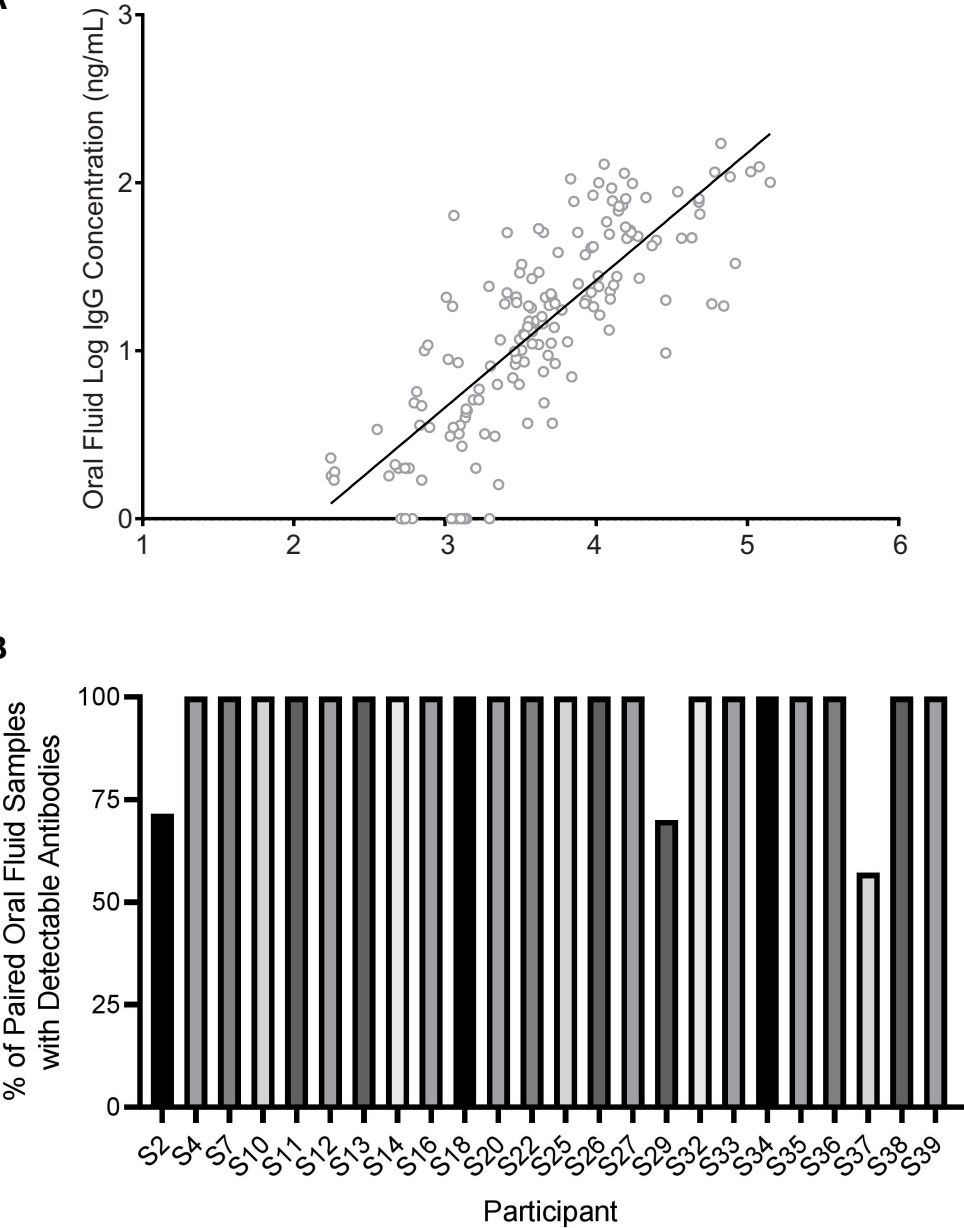
SARS-CoV-2 IgG in oral mucosal fluids of participants (n = 24) correlated with that of their serum. **(A)** Oral mucosal fluid IgG increased with serum IgG in the participants (R^2^ = 0.64, p < 0.0001). Each data point represents a single timepoint collection for both serum and oral mucosal fluids for a specific participant. The regression line is shown as the solid line. **(B)** Correlation between antibody detection in participants with paired serum and oral fluid specimens was typically high. On an individual basis, three individuals with detectable SARS-CoV-2 antibodies in serum (S2, S29, and S37) developed detectable oral mucosal fluid antibodies and maintained detectable antibody levels later in the time course.

Similar analyses (longitudinal trend and quantification) for antibody titers in serum can be found in the supplementary information (**Supplementary Figures 1**–**3**). The average IgG concentration found in the serum (11,601 +/- 21,227 ng/mL) of all participants throughout the study was higher than the average concentration found in oral mucosal fluids (29 +/- 33 ng/mL). A comparative analysis between serum and oral fluid seroconversion reveals consistent trends between the two specimen mediums (**Figure 4A**). IgG concentrations in the oral mucosal fluid positively correlated with the response in the serum of participants (R^2^ = 0.64, p < 0.0001).

Three individuals (S2, S29, S37) did not have a detectable antibody concentration in early oral mucosal fluid sample collections but developed a detectable concentration later in the study period (**Figure 4B**).

Interestingly, it was observed that these three individuals (S2, S29, S37) had lower concentrations of antibodies in serum (802, 1,000, and 500 ng/mL respectively) than the average observed IgG levels in other individuals (13629 +/- 22,564 ng/mL). Based on this small sample subset, we report that the oral mucosal fluid assay has a sensitivity of 87.5% at early time points. In another independent study (data not shown), we tested 60 paired serum and oral mucosal fluids samples from a cohort who were negative to SARS-CoV-2 infection and found the assay specificity to be 100%.

## Discussion

In this study, we have successfully monitored the persistence of IgG antibodies in serum and oral mucosal fluid specimens collected up to 12 months post symptom onset of SARS-CoV-2 infection. Furthermore, we developed a less invasive method to perform relative quantification of IgG against the S1 and S2 subunits in oral mucosal fluids that yields comparable results to the relative quantification of the S1 subunit of SARS-CoV-2 in serum. This is one of the first studies to longitudinally track and quantify antibody titers over a period of one year in response to SARS-CoV-2 infection using oral mucosal fluids with a serum-based assay as a comparator.

The change in antibody titer over the time course of the study was not found to be statistically significant in oral mucosal fluids, though we did observe an IgG titer asymptote around 6 months post-symptom onset. Our findings agree with two other recent studies showing the persistence of IgG in saliva specimens 60- and 115-days post-symptom onset (15, 16). In all specimens and sample types in the present study, antibodies that were originally detectable remained persistent throughout the timespan of the study (12-months), an encouraging finding which is consistent with other reports (5, 17, 18).

In a comparative analysis performed on a subset of our study cohort who received vaccination prior to the conclusion of the study, we found antibody concentration post-vaccination to be six-fold higher when compared to the mean maximum IgG concentration associated with natural infection. This result agrees with another research group which analyzed antibody response to Pfizer/BioNTech vaccination in serum in previously infected as well as COVID naïve healthcare workers (19). A recent work found anti-SARS-CoV-2 RBD IgG and neutralizing antibodies to be 10-100-fold higher following vaccination in 6 previously infected healthcare workers (20). In addition, they found that IgG titers in previously infected healthcare workers were similar at 21 days after their first vaccination and 7 days after their second vaccination. Another study found similar patterns of anti-SARS-CoV-2 spike protein IgG in a larger study cohort of healthcare workers from a medical center in Southern California (21). This suggests that previously infected individuals may only need one vaccine dose to confer adequate IgG titer compared to both doses in COVID naïve individuals, which may provide a strategy to maximize vaccine supply.

Though vaccination means that we can no longer quantify endogenous immunity following infection in those individuals, it is extremely reassuring to know that vaccination confers boosted antibody production. As more and more people become vaccinated, we are using the lessons learned from this study to monitor the persistence of antibodies in oral mucosal fluids against the Moderna or Pfizer vaccine in an ongoing year long clinical trial. Our early results are encouraging as we observe an increasing antibody titer in vaccinated participants consistent with what we observed in this cohort (10).

While our study provides critical insight into the persistence of IgG antibodies in both serum and oral mucosal fluid over a 12-month period, we believe our study size may be a major limitation. In earlier research, oral fluids and oral mucosal fluids proved to be a reliable specimen to monitor antibody response to various pathogens, including HIV and Hepatitis C. Sensitivity and specificity of the oral fluid-based assays were as follows: HIV [(98% & 98%) (22) and (99.3% & 99.8%) (13)], and Hep C [(89.9% & 100%) (23) and (98% & 98%) (24)]. Among oral fluids based assays, passive drool assays were less sensitive and specific than oral mucosal based assays such as OraSure. At some instances, oral mucosal fluids based assays could be less sensitive and specific compared to serum based assays. However, upon optimizing the assays for a specific pathogen, oral fluids provide remarkable flexibility in ease of collection, handling, cost, and importantly an improved sensitivity and specificity. While our SARS-CoV-2 antibody sensitivity is at 87.5% and specificity is at 100%, a smaller sample size is a source of concern. Further research is needed to validate the sensitivity and specificity parameters in a larger cohort.

Importantly, the relationship between the titers of S1 and S2 subunits of spike protein specific IgG antibodies and neutralization antibody titers need further research. As this understanding will enable us to use the S1/S2 antibody ELISA assay as a surrogate assay for complex virus neutralization assays. A recent report found a strong positive correlation between both anti-spike ectodomain and anti-RBD IgG titers and SARS-CoV-2 virus neutralization titers using convalescent plasma of 68 COVID-19 patients (25). This finding is consistent with other reports which found positive correlations between SARS-CoV-2 viral neutralization and S-RBD-specific IgG (26) as well as anti-S ELISA titers (27–29), warranting investigation of neutralization capacity for our current study cohort. Understanding the roles of S1/S2 antibodies as well as neutralization antibodies in a larger population will help us determine the role of these antibodies, and concentrations needed to confer long-term immunity against viral infection and reinfections.

Further research is needed on a larger sample set to help clarify these questions and to determine the correlation between IgG levels measured in this assay and the neutralizing antibody levels in oral mucosal fluids. Additionally, we were unable to evaluate the impact of other immune responses on protection against SARS-CoV-2 reinfection. It is known that T-cells play an important role in long term immune protection and therefore has been recently investigated in relation to SARS-CoV-2. A recent report found detectable IFN-γ secreting T cell responses in 71% (42/59) of recovered COVID-19 patients enrolled in their study (30). Similarly, SARS-CoV-2 specific CD8^+^ T cell responses have been shown to be important determinants of immune protection for individuals, including those with mild infection (25, 31, 32).

## Conclusion

Our current study provides critical insight into understanding the persistence of IgG antibodies in both serum and oral mucosal fluid over a 12-month period. However, an extended study period and a larger study cohort are needed to understand the following: a) long term persistence of antibodies in serum and oral mucosal fluids, b) characterize the difference in immune response to natural infection and vaccination, and c) understand the role of T-cells in individuals with and without antibody response. While vaccines provide much needed hope for ending this pandemic, full closure will be aided with an understanding of how long immunity persists and how frequently vaccination is needed. To accomplish this public health objective, we believe understanding the relationship between antibody levels and neutralization capacity to offer immune protection is critical. Through the establishment of a consistent, inexpensive, and non-invasive method for detection and relative quantification of SARS-CoV-2 antibodies in oral mucosal fluid specimens, communities will be provided an effective tool for making public health decisions to manage small outbreaks. Effective monitoring will enable identification and tracking of new variants *via* sequencing and the evaluation of antibody efficacy to neutralize the virus. Enforcement and availability of these tools will allow communities to better provide resources for treating infected individuals, reduce the spread of the virus, and return to pre-pandemic normalcy.

## Data Availability Statement

The original contributions presented in the study are included in the article/**Supplementary Material**. Further inquiries can be directed to the corresponding author.

## Ethics Statement

The study was initially approved by The UCLA Institutional Review Board (UCLA IRB) (IRB#20-000703). The UCLA IRB waived the requirement for signed informed consent for the research under 45 CFR 46.117(c) (2). The study team complied with all UCLA and Advarra IRB policies and procedures, as well as with all applicable Federal, State, and local laws regarding the protection of human subjects in research as stated in the approved IRB (Pro00045766). The patients/participants provided their written informed consent to participate in this study.

## Author Contributions

PC and AA designed and ran experiments, analyzed and interpreted data, and drafted the manuscript. MM designed the study protocol, designed experiments, analyzed and interpreted data, and drafted the manuscript. ND and MS analyzed and interpreted data, ran the experiments, and drafted the manuscript. RL, CB, and JC ran the experiments and organized data for processing. FT and VS conceptualized and designed the study protocol, designed experiments, and edited the manuscript. AI oversaw and designed experiments, oversaw data collection and analysis, and edited the manuscript. All authors contributed to the article and approved the submitted version.

## Funding

This study was funded by Curative.

## Conflict of Interest

PC, ANA, MAM, ND, AM, MS, RL, CB, JGC, KT, LL, NN, MB, FT, VS and AI were employed by the company Curative.

The authors declare that this study received funding from Curative. The funder had the following involvement with the study: employment of the abovementioned authors.

The remaining authors declare that the research was conducted in the absence of any commercial or financial relationships that could be construed as a potential conflict of interest.

## Publisher’s Note

All claims expressed in this article are solely those of the authors and do not necessarily represent those of their affiliated organizations, or those of the publisher, the editors and the reviewers. Any product that may be evaluated in this article, or claim that may be made by its manufacturer, is not guaranteed or endorsed by the publisher.
