# Supplementary material for: SARS-CoV-2 Specific IgG Antibodies Persist Over a 12-Month Period in Oral Mucosal Fluid Collected From Previously Infected Individuals
Source: Front Immunol. 2021 Dec 9;12:777858. doi: 10.3389/fimmu.2021.777858 (PMC8697108; doi:10.3389/fimmu.2021.777858)
Supplement: Supplementary file 1 [file DataSheet_1.pdf]

Supplementary Information

Serum IgG Response Cohort

The cohort (n=34) demographics for the serum IgG analysis including gender and age is presented in **Figure S1**. 13 individuals (38.2%) identified as male and 21 (61.8%) identified as female. During the time of collection, 8 individuals (23.5%) were younger than 25 years of age, 11 (32.4%) were between the ages of 25 and 40, and 15 individuals (44.1%) were older than 40 years of age. Of these participants, 38% (n =13) were not included in the original oral antibody dynamics analysis because they did not have adequate sample volume for quantification.

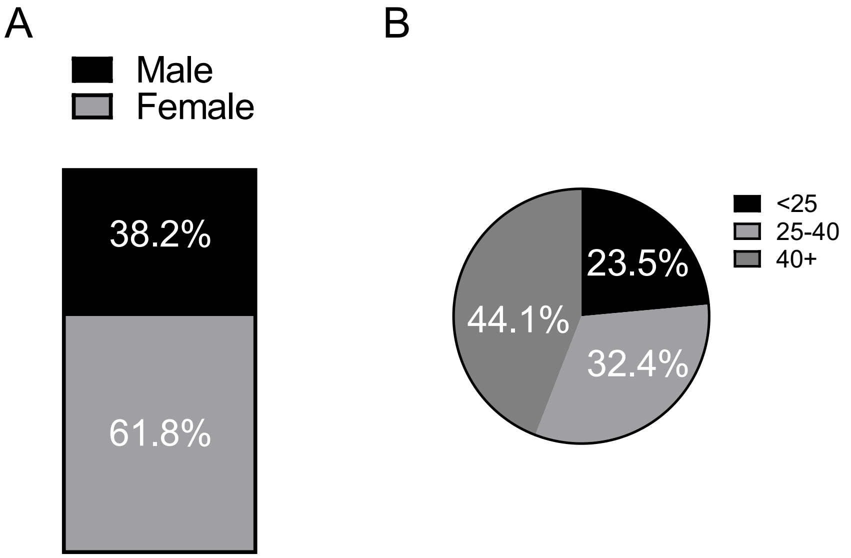

Figure S1: **Population demographics for serum cohort**. A population of 34 individuals were chosen who had contributed serum samples at timepoints spanning over 12-months for the clinical study. **A) Sex representation.** Of the 34 individuals whose serum samples were tested, 13 (38.2%) of them identified as male and 21 (61.8%) as female. **B) Age representation.** Age breakdowns are reported as we have done in previous studies. Eight (8) individuals (23.5%) were

younger than 25 years of age at the time of sample collection, 11 (32.4%) were between the ages of 25 and 40 and 15 individuals (44.1%) were older than 40 years of age.

Serum Analysis

For the serum study cohort (n=34), we assessed the longitudinal serum IgG antibody levels (**Supp. Fig. 2A**) that showed an exponential decline in IgG concentration from the peak IgG concentration (approx. 1.5 month). Also, to understand the IgG titer distribution, we calculated maximum IgG (IgG_{Max}) titer per participant, average IgG (IgG_{Avg}) per participant, and IgG concentration of the cohort around the 6-month period (IgG_{6month}) (**Supp. Fig. 2B**). We found that there was significant difference ($t=3.825$, $p=.0005$) in IgG levels between maximum IgG levels (IgG_{Max}) and IgG levels at 6 months (IgG_{6month}).

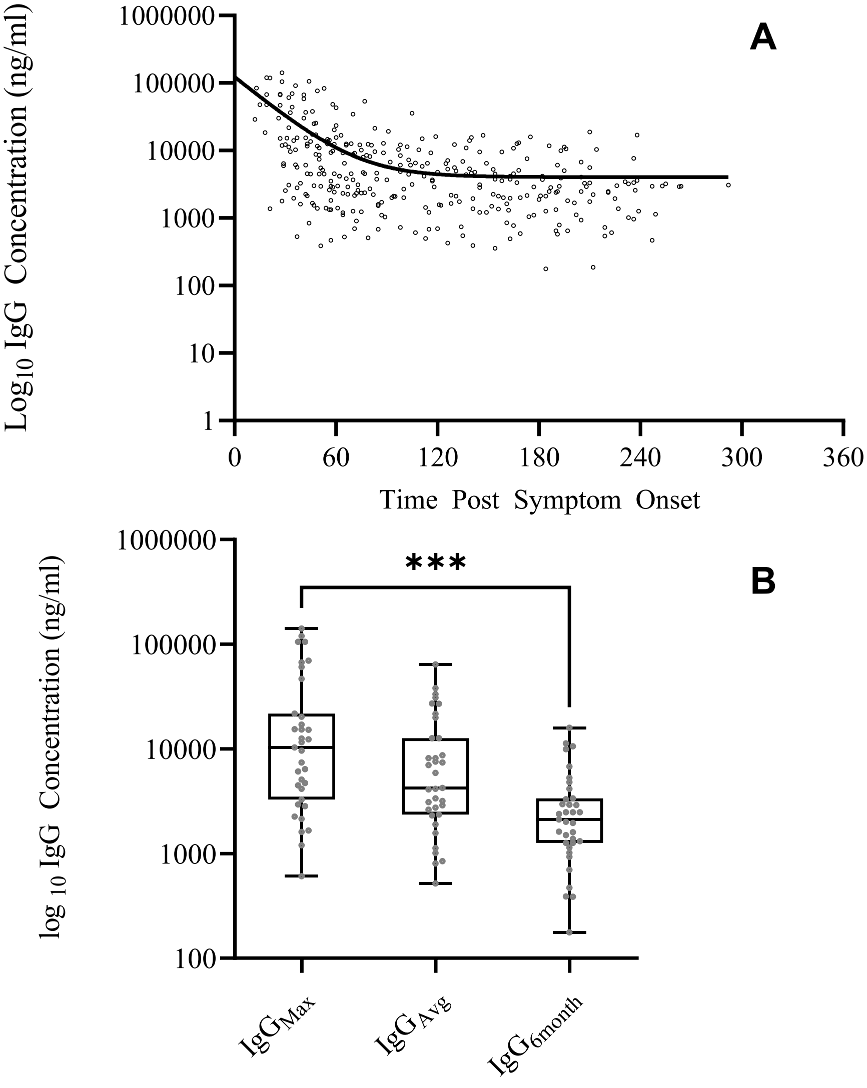

Figure S2: SARS-CoV-2 IgG antibodies were quantified in the serum of 34 individuals by ELISA. A) Quantification of SARS-CoV-2 antibodies in serum revealed a declining antibody titer over a period of six months following the peak concentration. From the peak titer up to 60 days (ng/mL), serum concentrations declined rapidly with time and followed an exponential decay. **B) A comparison of the maximum, average, and 6-month antibody concentration reveals a significantly decreasing**

antibody concentration. The maximum average IgG concentration (36149 +/- 44972 ng/mL) during the early immune response period was significantly higher than the average IgG concentration (4188+/- 4143 ng/mL) of the participants after 6 months, representing an 88% drop in antibody titer.
